# Supplementary material for: Acute kidney injury and its progression in hospitalized patients—Results from a retrospective multicentre cohort study with a digital decision support system
Source: PLoS One. 2021 Jul 12;16(7):e0254608. doi: 10.1371/journal.pone.0254608 (PMC8274880; doi:10.1371/journal.pone.0254608)
Supplement: S1 Table — (PDF) [file pone.0254608.s001.pdf]

S1 Table

Comparison of the frequency, the patient characteristics, the administrative coding, the case outcomes and the comorbidities between cases with progressive AKI and those without.

	**No AKI**	**AKIN 1** without progression	**AKIN1→AKIN2**	**AKIN 2** without progression	**AKIN1→AKIN3**	**AKIN2→AKIN3**	**AKIN 3** without progression
Frequency, n, %	70689, 87.9	6393, 8.0	920, 1.1	783, 1.0	740, 0.9	188, 0.2	676, 0.8
**Basic patient characteristics**							
Age (years), median [IQR] ***	67 [55-79]	73 [63-83]	69 [58.5-79.5]	71 [61-81]	69 [59-79]	67 [56.5-77.5]	72 [62-82]
Sex, % male **	54.4	56.2	55.2	55.2	66.8	59.0	63.2
First eGFR, median [IQR]	75 [53-97]	56 [32-80]	58 [35-81]	72 [50-94]	44 [18.5-69.5]	79 [55.5-102.5]	24 [0-57.5]
**Administrative coding**							
N17 coded, % ***	5.3	31.7	67.8	47.8	85.5	80.9	66.3
**Case outcomes**							
eGFR on discharge[#], median [IQR] ***	81 [60.5-101.5]	55 [32.5-77.5]	59 [36-82]	66 [40.5-91.5]	44 [19.5-68.5]	66 [38-94]	44 [12.5-75.5]
Total length of hospitalization (days), median [IQR] ***	7 [3-11]	14 [6-22]	23 [10.5-35.5]	15 [5-25]	26 [11-41]	22 [9.5-34.5]	15 [6-24]
Hospital mortality, % ***	2.3	15.6	41.0	26.3	51.5	47.3	28.7
Dialysis at least once, % ***	0.5	7.8	31.2	11.2	54.3	41.5	33.4

Dialysis within 72h of discharge, % ***	0.3	3.9	14.1	6.8	24.5	20.7	17.3
Intensive or intermediate care, % ***	13.1	26.6	36.2	22.0	38.5	34.6	24.9
**Nephrological consultation and time interval**							
Nephrological consultation at least once (ULMC only), % ***	0.1	1.7	4.1	1.5	8.0	8.5	7.4
Time from first AKI to first nephrological consultation (ULMC only), h, median	-	68.2	116.6	26.6	116.3	72.8	50.9
Time interval between first AKI and AKI max, h, median	-	-	48.5	-	73.6	26.1	-
**Comorbidities**							
Hypertension - I10., %	46.3	49.1	48.0	52.9	43.9	53.2	48.1
Diabetes mellitus - E11., %	25.5	36.1	34.9	34.2	38.5	31.4	38.0
Liver cirrhosis - K74., % ***	1.8	3.7	9.3	4.0	7.6	6.9	3.6
Coronary heart disease - I25., %	13.9	21.4	19.9	19.2	22.7	17.0	19.8
Myocardial infarction - I21., % ***	1.8	3.8	4.5	3.6	6.4	2.7	2.5
Cardiac insufficiency - I50., % ***	15.1	32.3	37.7	27.2	41.5	30.3	34.9
Exsiccosis - E86., %	5.0	6.3	6.3	7.7	6.2	4.3	12.7
Shock - R57., % ***	1.3	12.2	36.0	17.5	43.8	37.8	16.7

Sepsis - A41., % ***	3.0	16.0	37.3	22.6	43.6	39.4	24.4

Asterisks indicate significant differences between cases with AKI progression and cases without AKI progression (p-value *<0.05, **<0.01, ***<0.001).

#calculated from all patients who left the hospital alive and without dialysis within 72 h of discharge, given in ml/min/1.73m² (CKD-EPI formula)
